# Contraception use and HIV outcomes among women initiating dolutegravir‐containing antiretroviral therapy in Kenya: a retrospective cohort study

**DOI:** 10.1002/jia2.26046

**Published:** 2022-12-25

**Authors:** John M. Humphrey, Victor Omodi, Caitlin Bernard, Mercy Maina, Julie Thorne, Ann Mwangi, Kara Wools‐Kaloustian, Rena C. Patel

**Affiliations:** ^1^ Department of Medicine Indiana University School of Medicine Indianapolis Indiana USA; ^2^ Academic Model Providing Access to Healthcare (AMPATH) Eldoret Kenya; ^3^ Department of Obstetrics and Gynecology Indiana University School of Medicine Indianapolis Indiana USA; ^4^ Department of Obstetrics and Gynecology University of Toronto Toronto Ontario Canada; ^5^ Department of Behavioural Science School of Medicine Moi University Eldoret Kenya; ^6^ Departments of Medicine and Global Health Washington University Seattle Washington USA

**Keywords:** Africa, ARV, health policy, reproductive, teratogen, viremia

## Abstract

**Introduction:**

The rollout of dolutegravir (DTG) in low‐ and middle‐income countries was disrupted by a potential association reported with periconceptional DTG exposure among women living with HIV (WLHIV) and infant neural tube defects. This prompted countries to issue interim guidance limiting DTG use among women of reproductive potential to those on effective contraception. Data to understand the potential impact of such guidance on WLHIV are limited.

**Methods:**

We conducted a retrospective cohort analysis of WLHIV 15–49 years initiating DTG‐containing antiretroviral treatment (ART) in Kenya from 2017 to 2020. We determined baseline effective (oral, injectable or lactational amenorrhea) and very effective (implant, intrauterine device or female sterilization) contraception use among women who initiated DTG before (Group 1) or during (Group 2) the interim guideline period. We defined incident contraception use in each group as the number of contraceptive methods initiated ≤180 days post‐guideline (Group 1) or post‐DTG initiation (Group 2). We determined the proportions of all women who switched from DTG‐ to non‐nucleoside reverse transcriptase inhibitor (NNRTI)‐ (efavirenz or nevirapine) containing ART ≤12 months post‐DTG initiation, compared their viral suppression (<1000 copies/ml) and conducted multivariable logistic regression to determine factors associated with switching from DTG to NNRTI‐containing ART.

**Results:**

Among 5155 WLHIV in the analysis (median age 43 years), 89% initiated DTG after transitioning from an NNRTI. Baseline effective and very effective contraception use, respectively, by the group were: Group 1 (12% and 13%) and Group 2 (41% and 35%). Incident contraception use in each group was <5%. Overall, 498 (10%) women switched from DTG to an NNRTI. Viral suppression among those remaining on DTG versus switched to NNRTI was 95% and 96%, respectively (*p* = 0.63). In multivariable analysis, incident effective and very effective contraception use was not associated with switching.

**Conclusions:**

Baseline, but not incident, effective contraception use was higher during the interim guideline period compared to before it, suggesting women already using effective contraception were preferentially selected to initiate DTG after the guideline was released. These findings reveal challenges in the implementation of policy which ties antiretroviral access to contraceptive use. Future guidance should capture nuances of contraception decision‐making and support women's agency to make informed decisions.

## 1 INTRODUCTION

Since the recommendation by the World Health Organization (WHO) to include dolutegravir (DTG) as a preferred component of first‐ and second‐line antiretroviral treatment (ART) in 2018, DTG has been implemented in the majority of low‐ and middle‐income countries (LMICs) [1]. However, in May 2018, the early implementation of DTG in LMICs was disrupted by a report from Botswana signalling a possible association between periconception DTG exposure among women living with HIV (WLHIV) and incident neural tube defects (NTD) in their infants [2]. This report was immediately followed by interim guidance from the WHO and other regulatory agencies recommending that DTG use by women of reproductive potential be limited to those using consistent and reliable contraception [3, 4, 5]. Subsequent surveillance data indicated that the NTD risk was lower than initially reported, and potentially non‐significant [6, 7, 8]. In July 2019, DTG was recommended unequivocally by the WHO as a preferred agent for all WLHIV of reproductive potential regardless of contraception use [9]. Still, data to understand the real‐world impact of the NTD safety signal and ensuing WHO guidance on contraception use and DTG continuation are limited.

Furthermore, recent data suggest that the controversy surrounding the use of DTG among women of reproductive potential may have hindered its uptake in this population [1]. In Kenya and globally, a significant disparity in DTG uptake among women of reproductive potential compared to men of the same age range was observed [10]. In Uganda, a qualitative analysis found that its health systems were underprepared for the nuanced messaging and counselling required to implement DTG safely and effectively [11]. Many worried that the break in DTG use during the periconception period would drive increases in maternal HIV viremia, potentially worsening outcomes for women, their pregnancies and their infants [12]. Contraceptive uptake and HIV outcomes during this period of changing DTG guidance are important and remain unanswered.

Kenya was among the first countries in sub‐Saharan Africa to implement DTG. In October 2017, the Kenya Ministry of Health (MoH) piloted the implementation of DTG as a component of first‐line ART, which included women of reproductive potential. The Academic Model Providing Access to Healthcare (AMPATH), a large USAID‐funded HIV care and treatment programme in western Kenya, was among the initial programmes selected to lead the country's DTG rollout. Hence, DTG and contraception use at AMPATH before, during and after changing guidelines presents a unique opportunity to fill the knowledge gap concerning contraceptive use and HIV outcomes for women in LMICs after the NTD safety signal. Thus, we conducted a mixed methods study, named *Chaguo Langu* or “My Choice,” to better understand patient and provider perspectives and outcomes. In the present analysis, our primary objective was to determine whether there was an increase in the incidence of effective contraception use among WLHIV receiving DTG‐containing ART after the WHO interim guidance. We hypothesized that such an increase would be observed. As a secondary objective, we assessed the characteristics and early outcomes of those who switched from DTG‐ to non‐nucleoside reverse transcriptase inhibitor‐ (NNRTI) containing ART within 12 months of DTG initiation.

## 2 METHODS

### 2.1 Study design, setting and population

For this retrospective cohort study, we used electronic medical record data prospectively collected from WLHIV enrolled in HIV care at public‐sector health facilities supported by the AMPATH programme from 2017 to 2020. AMPATH provides standard‐of‐care HIV services based on national guidelines to more than 150,000 patients and participates in the International Epidemiology Databases to Evaluate AIDS consortium [13, 14, 15, 16, 17]. At the time of the study, routine viral load (VL) testing was recommended according to national and WHO guidelines, at 6 months after ART initiation, and if <1000 copies/ml, every 6 months for individuals and annually for those <25 and ≥25 years, respectively [18, 19]. Individuals with a VL ≥1000 copies/ml were recommended to have the test repeated after ≥3 months of enhanced adherence counselling and support.

The study was approved by the Institutional Research and Ethics Committee at Moi University and Moi Teaching and Referral Hospital in Kenya and the University of Washington Human Subjects Division in the United States. Based on the use of de‐identified data, the study was deemed exempt from IRB review at Indiana University, United States. Patient‐level consent was waived by the regulatory bodies because the data were collected as part of routine care and de‐identified. The reporting of this study conforms to the STROBE guidelines [20].

### 2.2 DTG policy in the context of the study

Prior to the implementation of DTG in Kenya, efavirenz‐containing ART was the recommended first‐line regimen for adults living with HIV. In October 2017, when Kenya MoH launched a broad rollout of DTG‐containing ART, AMPATH disseminated the MoH guidance to its clinicians through training programmes, medical education seminars and WhatsApp social media groups. Within a month after the WHO interim guidance, the Kenya MoH issued a circular recommending efavirenz‐containing ART as the preferred first‐line regimen for women of reproductive potential, and that women of reproductive potential receiving DTG‐containing ART switch to efavirenz‐containing ART [21]. In August 2018, the Kenya MoH issued guidelines recommending that “women and adolescent girls of childbearing potential who are currently on DTG should be counselled on the potential risk of NTD if she becomes pregnant and offered effective contraception” [15]. Updated WHO guidelines recommending the use of DTG among women of reproductive potential regardless of contraception use were issued in 2019, and similar updates were released by the Kenya MoH and AMPATH shortly thereafter [22].

### 2.3 Data management

We used clinical data available in the AMPATH electronic medical record that were collected by clinicians during routine care [23]. Inconsistencies in documented ART regimens were resolved through medical record review.

### 2.4 Cohort selection

We included WLHIV in the analysis if they were enrolled in HIV care at an AMPATH‐supported facility, initiated DTG‐containing ART on or after 1 October 2017, 15–49 years of age (inclusive) at the time of DTG initiation, and initiated DTG ≥12 months prior to 6 February 2020 (database closure). We restricted our analysis to women 15–49 years of age in accordance with the WHO definition of women of reproductive potential [24]. We set a 12‐month window after DTG initiation to allow sufficient time to observe viral suppression in the context of AMPATH's routine VL monitoring schedules [15].

### 2.5 Primary outcome

Our primary outcome was the incidence of very effective or effective contraception use among WLHIV on DTG after the WHO interim guideline. Contraception methods were classified according to their effectiveness: very effective (implant, intrauterine device or female sterilization), effective (oral, injectable or lactational amenorrhea), moderately effective (male condom and rhythm method), less effective (female condom or diaphragm) and any method (i.e. one or more of the above methods) [24].

**Figure 1:**
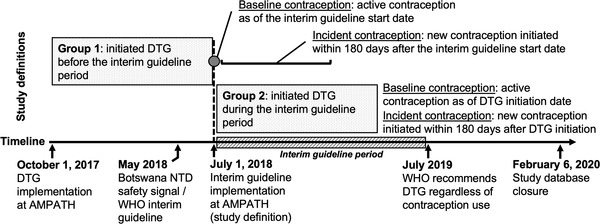
Contraception outcomes for Groups 1 and 2 in the context of the implementation of DTG and contraception policy in Kenya, for *Chaguo Langu* study, 2017–2020.

To determine contraception use, we disaggregated women into two groups based on whether they initiated DTG before (Group 1) or during (Group 2) the interim guideline period (Figure 1). To allow time for local dissemination of the guideline, we set 1 July 2018 as the start date for the interim guideline period in our analysis. For Group 1, baseline contraception was defined as the point prevalence of contraception used by women as of the interim guideline start date; incident contraception use was defined as the number of new contraceptive methods initiated within 180 days on or after the interim guideline start date among women not already using very effective contraception. For Group 2, baseline contraception was defined as the point prevalence of contraception used by women as of their DTG initiation date; incident contraception use was defined as the number of new contraceptive methods initiated within 180 days after their DTG initiation date among women not already using very effective contraception. Only women who remained on DTG for at least 180 days were included in each group. Importantly, the eligibility requirement that women initiate DTG at least 12 months before the database closure date ensured that the updated WHO guidance recommending DTG for all WLHIV (i.e. the time after the interim guideline period) did not overlap with the assessment window for incident contraception use among WLHIV in Group 2.

To assess whether women initiated contraception *before* initiating DTG (e.g. as a lead‐in to DTG initiation during the interim guideline period), we also determined incident contraception use using a window of 180 days before and after the date of DTG initiation, stratified by 3‐month periods beginning on the start date of the DTG rollout at AMPATH. The denominator and numerator in each period were the number of women who initiated DTG and contraception during the period, respectively.

### 2.6 Secondary outcome

Our secondary outcome was viral suppression (<1000 copies/ml) among all women receiving a DTG‐containing, first‐line regimen for ≥10 weeks, as well as among women who subsequently switched from DTG to an NNRTI (efavirenz or nevirapine)‐containing regimen within 12 months after DTG initiation. We selected for analysis the first VL result recorded ≥10 weeks after DTG initiation or switch to NNRTI, regardless of its timing with the interim guideline.

### 2.7 Covariates

We assessed the following covariates at the time of DTG initiation: age, weight, marital status, number of children, years in school, WHO Clinical Stage, CD4 cell count at ART initiation, ART regimen before DTG initiation, antiretrovirals co‐administered with DTG, years on ART, pregnancy status and receipt of treatment for active tuberculosis. We assessed viral suppression before DTG initiation by selecting the nearest VL ≤6 months before DTG initiation among individuals already on ART for ≥6 months [15]. Additional variables assessed at 12 months after DTG initiation were: the proportion of patients who switched to another regimen, transfer out, death and attrition, defined as missing the last scheduled visit before the 12‐month anniversary by >30 days. For each variable, we selected the nearest value within 180 days before and after each time point.

### 2.8 Statistical analysis

We calculated frequencies and proportions for categorical variables and medians and interquartile ranges (IQR) for continuous variables at DTG initiation and 12 months DTG initiation. The characteristics of Groups 1 and 2 were compared using 95% confidence intervals (CI) and Chi‐square tests for categorical and Wilcoxon rank‐sum for continuous measures.

To determine the primary outcome, we compared incident and baseline contraception in Groups 1 and 2, respectively, using two‐sided Chi‐square and Fisher's exact tests as appropriate. To control for potential confounding factors associated with a change in baseline very effective or effective contraception use during compared to before the interim guideline period, we used multivariate logistic regression models in which time zero was the date of DTG initiation and the primary predictor was Group 1 versus Group 2, adjusted for age, marital status, years in school and years on ART.

For the secondary outcome, we conducted multivariate logistic regression modelling to assess factors associated with a switch to NNRTI‐containing ART within 12 months after DTG initiation. We included the following *a priori* covariates at DTG initiation: age, marital status, years in school, weight, WHO Clinical Stage, newly initiated ART (i.e. initiated DTG as part of the first regimen vs. not), ART regimen at DTG initiation (i.e. tenofovir/lamivudine/DTG vs. any other DTG‐containing regimen), years on ART at DTG initiation and baseline or incident use of very effective or effective contraception. Analyses were conducted using SAS version 9.4 (SAS Institute, Cary, North Carolina, USA) and R version 3.6.2.

## 3 RESULTS

Among 12,613 WLHIV 15–49 years of age who initiated DTG‐containing ART at AMPATH, 7458 were excluded because they initiated DTG <12 months prior to database closure, resulting in 5155 women included in the analysis (Figure S1). The characteristics of WLHIV included in the analysis were similar to those excluded from the analysis (Table S1).

### 3.1 Characteristics at DTG initiation and 12 months after DTG initiation

The median (IQR) age of women included in the analysis was 43 (38–46) years, 61% had WHO Clinical Stage 1 or 2 disease and their median time on ART was 8 (4–11) years (Table 1). Most women (4719, 92%) initiated DTG after transitioning from another regimen, while 436 (9%) newly initiated DTG as part of their first regimen. Among those who transitioned to DTG from another regimen, 4570 (97%) transitioned from a first‐line NNRTI‐containing regimen. Tenofovir disoproxil plus lamivudine was co‐prescribed with DTG for 4701 (91%) women. The remaining 454 (9%) women initiated DTG along with other antiretrovirals, most commonly other nucleoside reverse transcriptase inhibitors (e.g. abacavir or zidovudine in 96% of the subset). An eligible VL before DTG initiation was available for 3482 (74%) of 4719 women who transitioned to DTG from another regimen, among whom 3368 (97%) were virally suppressed. The characteristics of women in Groups 1 and 2 were generally similar.

**Table 1:** Characteristics of WLHIV at DTG initiation for *Chaguo Langu* study, 2017–2020

Characteristic	Total *N* = 5155 *n* (%)	Group 1^a^ *N* = 2065 *n* (%)	Group 2^a^ *N* = 2288 *n* (%)	*p*‐Value
Age, median years (IQR)	43 (38–46)	42 (38–46)	44 (40–47)	<0.001
Weight, median kg (IQR)^b^	62 (54–72)	64 (55–74)	62 (54–72)	<0.001
Married or cohabitating^c^	2215 (45)	942 (47)	999 (46)	0.38
Number of children, median (IQR)^d^	3 (2–4)	3 (2–4)	3 (2–5)	<0.001
Years of school, median (IQR)^e^	8 (7–12)	8 (7–12)	8 (7–12)	0.001
CD4, median cells/mm^3^ (IQR)^f^	359 (138–589)	473 (320–688)	342 (170–552)	0.02
WHO Stage				
Stage 1 or 2	3124 (61)	1309 (64)	1449 (63)	0.53
Stage 3 or 4	1576 (30)	666 (32)	707 (31)	
Missing	455 (9)	90 (4)	132 (6)	
Years on ART, median (IQR)	8 (4–11)	7 (5–11)	9 (5–11)	<0.001
Newly initiated ART	436 (9)	109 (5)	148 (6)	0.10
Antiretroviral base pre‐DTG initiation^g^				
NNRTI	4570 (97)	1920 (97)	2124 (97)	0.47
Other^h^	149 (3)	60 (3)	58 (3)	
ART regimen at DTG initiation				
TDF + 3TC + DTG	4701 (91)	1790 (87)	2024 (88)	0.08
Other^i^	454 (9)	275 (13)	264 (12)	
Pregnant	78 (2)	31 (2)	17 (1)	0.02
On treatment for active tuberculosis	112 (2)	33 (2)	32 (1)	0.59
Viral suppression pre‐DTG initiation^j^	3368 (97)	1364 (97)	1653 (98)	0.002

Abbreviations: 3TC, lamivudine; ART, antiretroviral treatment; DTG, dolutegravir; IQR, interquartile range; kg, kilogram; NNRTI, non‐nucleoside reverse transcriptase inhibitor; TDF, tenofovir disoproxil; WHO, World Health Organization.

^a^ The total patients in Groups 1 and 2 do not equal 5155 because of the criteria that women in each group remain on DTG for at least 180 days.

^b^ *n* = 5016.

^c^ *n* = 4937.

^d^ *n* = 4792.

^e^ *n* = 3770; the significant *p*‐value of 0.001 despite identical medians is attributable to rounding of the median and IQR values.

^f^ *n* = 197.

^g^ Denominator is 4719 WLHIV who initiated ART prior to DTG initiation.

^h^ Includes atazanavir or lopinavir (*n* = 132), raltegravir (*n* = 6), darunavir (*n* = 5) and dual base class (*n* = 6).

^i^ Includes one or more of the following: abacavir or zidovudine (*n* = 435), atazanavir or lopinavir (*n* = 21), darunavir (*n* = 10) and etravirine (*n* = 3).

^j^ Denominator is 3482 WLHIV with an eligible viral load.

At 12 months after DTG initiation, 4498 (87%) women had continued a DTG‐containing regimen and 657 (13%) had switched to a non‐DTG‐containing regimen (Table S2). Among those who switched, 604 (92%) switched to an NNRTI‐containing regimen. The median time on DTG prior to the switch was approximately 5 months. Attrition and death occurred in 293 (6%) and 54 (1%) women, respectively.

### 3.2 Primary outcome: contraception use

Among 2065 women in Group 1 at baseline, 1560 (76%) were using any contraception (71% reported male condom use), 12% were using effective contraception and 13% were using very effective contraception according to WHO classifications (Table 2). Among 2288 women at baseline in Group 2, 2145 (94%) were using any contraception (91% reported male condom use), 41% were using effective contraception and 35% were using very effective contraception. For Group 2, the median (IQR) time from baseline very effective contraception initiation to DTG initiation was 4 (2–7) years, and 7 (5–9) years for effective contraception, well before the interim guideline was released. Incident contraception use was <5% in Groups 1 and 2 across each contraceptive method effectiveness category.

**Table 2:** Baseline and incident contraception use among WLHIV who initiated DTG before (Group 1) and during (Group 2) the interim guideline period, for *Chaguo Langu* study, 2017–2020

	Baseline contraception use			Incident contraception use		
Contraception method^a^	Group 1 *N* = 2065 *n* (%)	Group 2 *N* = 2288 *n* (%)	*p*‐value	Group 1 *N* = 1803 *n* (%)	Group 2 *N* = 1491 *n* (%)	*p*‐value
Any method	1560 (76)	2145 (94)	<0.001	38 (2.1)	123 (8)	<0.001
Very effective	262 (13)	797 (35)	<0.001	9 (0.5)	42 (3)	<0.001
Implant	161 (8)	441 (19)	<0.001	7 (0.4)	24 (2)	<0.001
Intrauterine device	28 (1)	291 (13)	<0.001	2 (0.1)	9 (0.6)	0.03
Female sterilization	62 (3)	216 (9)	<0.001	1 (<0.1)	10 (0.7)	0.004
Effective	253 (12)	942 (41)	<0.001	4 (0.2)	14 (0.9)	0.01
Oral	34 (2)	221 (10)	<0.001	0 (0)	3 (0.2)	0.09
Injectable	215 (10)	855 (37)	<0.001	4 (0.2)	10 (0.7)	0.06
Lactational amenorrhea	1 (<0.1)	4 (0.2)	0.38	0 (0)	1 (<0.1)	0.45
Moderately effective	1470 (71)	2090 (91)	<0.001	33 (2)	101 (7)	<0.001
Male condom	1470 (71)	2090 (91)	<0.001	33 (2)	101 (7)	<0.001
Rhythm method	0 (0)	75 (3)	<0.001	0 (0)	0 (0)	–
Less effective	0 (0)	22 (1)	<0.001	0 (0)	0 (0)	–
Female condom or diaphragm	0 (0)	21 (1)	<0.001	0 (0)	0 (0)	–

^a^ Contraceptive effectiveness is measured by the number of pregnancies per 100 women using the method per year: very effective (0–0.9 pregnancies per 100 women); effective (1–9 pregnancies per 100 women); moderately effective (10–19 pregnancies per 100 women); and less effective (20 or more pregnancies per 100 women). The totals for each effectiveness class differ from the sum of the methods within the class due to some women being on two or more methods with no documented stop date.

**Figure 2:**
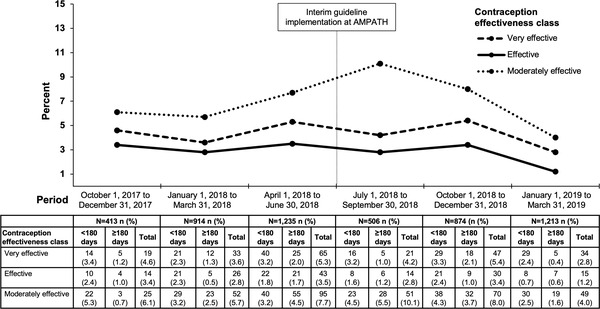
Incident contraception use within 180 days before and after DTG initiation among women initiating DTG during 3‐month periods from 2017 to 2019, stratified by contraception effectiveness class.

Incident very effective or effective contraception use within 180 days before and after DTG initiation also remained ≤5% across periods from 2017 to 2019, with no increase during the interim guideline period (Figure 2). Additionally, women with incident use of very effective or effective contraception, compared to other women, had a lower median age (30 vs. 40 years, *p*<0.001) and a higher proportion had newly initiated ART (85% vs. 7%, *p*<0.001) (Table S3). In multivariable analysis, DTG initiation during the interim guideline period (i.e. Group 2) was associated with higher odds of baseline very effective or effective contraception use compared to DTG initiation before the interim guideline period (i.e. Group 1), along with being married/cohabitating, and a higher number of years on ART (Table S4). Older age and having a higher number of years in school were associated with slightly lower odds.

### 3.3 Secondary outcome: viral suppression

A total of 4543 women continued DTG for ≥10 weeks and had a VL available ≥10 weeks after DTG initiation (FigureS2). Of these, 439 (10%) switched to an NNRTI within 12 months after DTG initiation and 376 (86%) of them had a VL available ≥10 weeks after switching to an NNRTI (Figure S3). Viral suppression was similar among women who continued DTG compared to women after they switched to an NNRTI (95% vs. 96%, *p* = 0.63). In multivariable analysis, older age and being on tenofovir/lamivudine/DTG compared to other DTG‐containing regimens were associated with higher odds of switching, being married/cohabitating was associated with lower odds and effective contraception use was not associated with switching (Table S5). A sensitivity analysis including those who “newly initiated ART” yielded results similar to the primary analysis (Table S6).

## 4 DISCUSSION

In our study, we found no increase in incident very effective or effective contraception use among reproductive‐aged women receiving DTG‐containing ART during the interim guideline period compared to before the guideline was released. Multiple factors influence contraception use and decision‐making, including a woman's knowledge and preferences, her and her partner's current and future reproductive plans, provider biases, access to contraceptive services and institutional, social, and cultural norms [25]. These factors pose a fundamental challenge to measuring contraception use as an indicator of policy adherence.

The low incidence of contraception use during the interim guideline period may have been driven, in part, by women's perceptions about contraception or their partners’ or providers’ influence on contraceptive decision‐making [26]. The median age of women in our study was 43 years, compared to 30 years for the subset of women who initiated very effective or effective contraception. Older women, or their providers, may have perceived lower risk of pregnancy because they were near the end of their reproductive potential and elected to continue DTG without effective contraception despite the potential NTD risk.

Baseline use of very effective or effective contraception was also higher among women who initiated DTG during the interim guideline period compared to before it. This finding probably suggests that women already using effective contraception were preferentially selected to initiate DTG. This finding also helps elucidate why in our prior research we identified lower DTG uptake among women of reproductive age compared to other groups, that is fewer women met the highly selective contraceptive criteria for DTG initiation [10]. Our finding is also consistent with our qualitative research which showed that providers were aware of the interim guideline but felt underprepared to navigate the complex decision‐making for reproductive health that it required [27]. Additional decision support tools, which were strongly desired by the providers, would help them implement nuanced interim guidelines for WLHIV in the future.

At the programmatic level, the lack of contraception uptake may reflect the need to enhance communication platforms and access to contraceptive services. Integrating family planning and HIV services, available in some but not all AMPATH clinics, is a proven strategy to enhance women's access to contraception and reproductive healthcare [28].

We found that 13% of women initiating DTG switched off it, and that switching from DTG was not associated with baseline or incident use of very effective or effective contraception. This finding further sharpens the complex picture of the programmatic response to the interim guidance, which largely failed to help more women initiate an effective contraception method. The reasons underlying switches are also likely multifold, including DTG‐related adverse effects, and women's individual preferences, reproductive plans or perceived risk of pregnancy [29, 30, 31, 32]. The adjusted odds of switching to an NNRTI were higher among those being on a tenofovir, lamivudine and DTG regimen compared to women on alternative regimens, suggesting that the latter group may have had additional indications to maintain DTG, such as antiretroviral tolerability or suspected HIV drug resistance. More research is needed to understand the frequency and drivers of DTG discontinuations, regardless of the NTD issue, in LMICs.

Finally, viral suppression was similarly high among women who remained on DTG and those who switched from DTG to an NNRTI. Meta‐analyses and modelling studies have shown superior viral suppression with DTG compared to efavirenz‐containing regimens [33, 34, 35, 36]. However, two recent trials in LMICs showed equivalent virologic suppression between DTG and efavirenz [37, 38]. We suspect that the women selected to initiate DTG in our study were well‐engaged in care and had excellent historical adherence to NNRTI‐containing ART, which enabled them to maintain viral suppression on either regimen.

Our study is strengthened by its analytical approach which leverages real‐world, robust electronic medical record data from one of the largest HIV care programmes in Africa. However, as with all observational studies, our analyses are subject to residual and unobserved confounding. Specifically, women's reproductive intentions (unavailable in our data) may have strongly impacted their contraception and DTG decisions. It is also possible that the interim guideline influenced contraception dynamics beyond incidence, such as more consistent contraception use among women (not ascertainable in our study). The date we used to define the start of the interim guidance may not reflect the likely more gradual and heterogeneous implementation of the guidance in our setting. However, the broad windows used to define incident contraception uptake likely account for this variability. While the initiation of effective contraception use in Group 2 could be misclassified as baseline use, when they actually represented early contraception initiation in response to the NTD safety signal, this misclassification is unlikely because most women in Group 2 initiated effective contraception years prior to the interim guideline release. It is possible that the increase in baseline contraception use during the interim guideline period may reflect enhanced clinician documentation of contraception use rather than contraception uptake. Notwithstanding these limitations, our findings are consistent with prior studies identifying gaps in programme‐wide responsiveness to the interim guidance for WLHIV in East Africa [10, 11].

## 5 CONCLUSIONS

Baseline, but not incident, effective contraception use was higher during the interim guideline period compared to before it, suggesting women already using effective contraception were preferentially selected to initiate DTG after the guideline was released. Our findings reveal inherent challenges in the implementation of public health policy which ties antiretroviral access to contraceptive use. Contraceptive decision‐making is influenced by a complex myriad of factors, both within and outside of patient–provider dyads. Policy pivoting on contraception use could also exacerbate existing disparities in treatment access among WLHIV and worsen reproductive health justice for these women. Interventions to address contraception use among WLHIV should focus on supporting women's agency to make informed healthcare decisions with their providers and developing public health guidance which appropriately captures the nuances of contraception decision‐making and women's right to lead it.

## COMPETING INTERESTS

The authors declare that they have no competing interests.

## AUTHORS’ CONTRIBUTIONS

JMH, RCP, CB and MM conceptualized and designed the study and had primary responsibility for the interpretation of the data. JH, VO, CB, JT and MM were responsible for regulatory approval and data collection. VO and AM analysed the data. JH wrote the paper with assistance from all co‐authors. All authors have read and approved the final manuscript.

## FUNDING

Dr. John M. Humphrey's effort was supported by the Eunice Kennedy Shriver National Institute of Child Health & Human Development (NICHD, K23HD105495). Dr. Rena C. Patel's effort and data analysis was supported by the National Institute of Allergy and Infectious Diseases (NIAID, K23AI120855). This work was also supported by intramural funds provided to Dr. Humphrey by Indiana University.

## DISCLAIMER

The content is solely the responsibility of the authors and does not necessarily represent the official views of the National Institutes of Health.

## Supporting information

Supporting information
**Figure S1**. Eligibility flow diagram for *Chaguo Langu* study, 2017–2020.
**Figure S2**. Viral suppression among women who continued DTG during the 12 months after DTG initiation.
**Figure S3**. Viral suppression among women who switched from DTG to efavirenz or nevirapine during the 12 months following DTG initiation.
**Table S1**. Characteristics of patients included in the analysis and those excluded because they initiated DTG <12 months prior to the date of database closure.
**Table S2**. Characteristics of WLHIV during the 12 months following DTG initiation for *Chaguo Langu* study, 2017–2020.
**Table S3**. Comparison of WLHIV in Groups 1 and 2 who were on effective or very effective contraception at baseline, those with incident use of effective or very effective contraception, and all other WLHIV.
**Table S4**. Unadjusted and adjusted odds ratios for factors associated with baseline very effective or effective contraception use among WLHIV.
**Table S5**. Characteristics of women at DTG initiation, categorized by DTG continuation versus switch to NNRTI within 12 months after initiating DTG, and associations with the switch to NNRTI for *Chaguo Langu* study, 2017–2020.
**Table S6**. Sensitivity analysis to determine associations with the switch to NNRTI, including (Model A) and excluding (Model B) the variable “newly initiated ART” (*n* = 4480) and excluding the variable “Very effective or effective contraception use.”

## Data Availability

The data that support the findings of this study are available from the corresponding author upon reasonable request.
