# Supplementary material for: Contraception use and HIV outcomes among women initiating dolutegravir‐containing antiretroviral therapy in Kenya: a retrospective cohort study
Source: J Int AIDS Soc. 2022 Dec 25;25(12):e26046. doi: 10.1002/jia2.26046 (PMC9790976; doi:10.1002/jia2.26046)
Supplement: Supplementary file 1 — Supporting information Figure S1. Eligibility flow diagram for Chaguo Langu study, 2017–2020. Figure S2. Viral suppression among women who continued DTG during the 12 months after DTG initiation. Figure S3. Viral suppression among women who switched from DTG to efavirenz or nevirapine during the 12 months following DTG initiation. Table S1. Characteristics of patients included in the analysis and those excluded because they initiated DTG <12 months prior to the date of database closure. Table S2. Characteristics of WLHIV during the 12 months following DTG initiation for Chaguo Langu study, 2017–2020. Table S3. Comparison of WLHIV in Groups 1 and 2 who were on effective or very effective contraception at baseline, those with incident use of effective or very effective contraception, and all other WLHIV. Table S4. Unadjusted and adjusted odds ratios for factors associated with baseline very effective or effective contraception use among WLHIV. Table S5. Characteristics of women at DTG initiation, categorized by DTG continuation versus switch to NNRTI within 12 months after initiating DTG, and associations with the switch to NNRTI for Chaguo Langu study, 2017–2020. Table S6. Sensitivity analysis to determine associations with the switch to NNRTI, including (Model A) and excluding (Model B) the variable “newly initiated ART” (n = 4480) and excluding the variable “Very effective or effective contraception use.” [file JIA2-25-e26046-s001.docx]

**Supporting Information**

**S1 Table.** Characteristics of patients included in the analysis and those excluded because they initiated DTG <12 months before the date of database closure.

| **Characteristic** | **Included in analysis**  **N=5,155**  **n (%)** | **Excluded because they initiated DTG <12 months before database closure**  **N=7,458**  **n (%)** | **P-value**^a^ |
| --- | --- | --- | --- |
| Age at DTG initiation, median years (IQR) | 43 (38-46) | 43 (37-46) | <0.001 |
| Age at ART initiation, median years (IQR) | 36 (32-39) | 35 (30-40) | <0.001 |
| CD4 count at ART initiation, median cells/mm^3^ (IQR) | 359 (138-589) | 300 (168-475) | <0.001 |
| Time on ART at DTG initiation, median years (IQR) | 7.5 (4.2-11) | 6.2 (2.2-10) | <0.001 |

^a^ Kruskal-Wallis Test

**S2 Table.** Characteristics of WLHIV during the 12 months following DTG initiation for *Chaguo Langu* study, 2017-2020.

| **Characteristic** | **N=5,155**  **n (%)** |
| --- | --- |
| Continued DTG | 4,498 (87) |
| Switched off DTG | 657 (13) |
| Switched to NNRTI^a^ | 604 (92) |
| Switched to PI^b^ | 48 (7) |
| Other^c^ | 5 (0.8) |
| Time on DTG prior to switch, median months (IQR) |  |
| Switch to NNRTI | 5 (3-8) |
| Switch to PI | 6 (2-8) |
| Weight change at 12 months, median kg (IQR)^c^ | 1 (-1 to 4) |
| Transfer out | 66 (1) |
| Attrition | 293 (6) |
| Death | 54 (1) |

ART, antiretroviral treatment; DTG, dolutegravir; IQR, interquartile range; kg, kilogram; NNRTI, non-nucleoside reverse transcriptase inhibitor; PI, protease inhibitor

^a^559 switched to efavirenz and 45 to nevirapine; ^b^47 switched to atazanavir or lopinavir, one switched to darunavir; ^c^One switched to raltegravir, 4 with no base class documented; ^d^n=3,803

**S3 Table.** Comparison of WLHIV in Groups 1 and 2 who were on very effective or effective contraception at baseline, those with incident use of very effective or effective contraception, and all other WLHIV.

| **Characteristic** | **Baseline very effective or effective contraception use**  **N=2,122**  **n (%)** | **Incident very effective or effective contraception use**  **N=63**  **n (%)** | **No baseline or incident use of very effective or effective contraception**  **N=2,168**  **n (%)** | **P-value** |
| --- | --- | --- | --- | --- |
| Age at DTG initiation, median years (IQR) | 42 (38-45) | 30 (36-40) | 40 (40-47) | <0.001^a^ |
| Newly initiated ART | 48 (2.3) | 54 (85) | 155 (7) | <0.001^b^ |
| Time on ART at DTG initiation, median years (IQR) | 8.4 (5.9-11) | 0 (0-0.1) | 7.7 (4.6-11) | <0.001^a^ |

^a^ Kruskal-Wallis Test; ^b^ Mantel-Haenszel chi-square

**S4 Table.** Unadjusted and adjusted odds ratios for factors associated with baseline very effective or effective contraception use among WLHIV.

| **Characteristic** | **N** | **Unadjusted OR (95% CI)** | **Adjusted OR (95% CI)** |
| --- | --- | --- | --- |
| Age (years) at DTG initiation^a^ | 4,290 | 0.95 (0.94-0.96) | 0.91 (0.90-0.93) |
| Married or cohabitating | 4,143 |  |  |
| No |  | Ref. | Ref. |
| Yes |  | 1.81 (1.60-2.04) | 1.91 (1.66-1.44) |
| Years in school | 3,409 | 0.96 (0.94-0.98) | 0.97 (0.95-0.99) |
| Years on ART at DTG initiation | 4,290 | 1.06 (1.04-1.07) | 1.05 (1.02-1.07) |
| Timing of DTG initiation | 4,290 |  |  |
| Group 1 |  | Ref. | Ref. |
| Group 2 |  | 1.48 (1.31-1.67) | 2.10 (1.81-2.44) |

^a^ N=4,290 and not 4,293 because this analysis does not include the 63 women classified as incident very effective or effective contraception use.

**S5 Table.** Characteristics of women at DTG initiation, categorized by DTG continuation versus switch to NNRTI within 12 months after initiating DTG, and associations with switch to NNRTI for *Chaguo Langu* study, 2017-2020.

| **Characteristic** | **N** | **DTG continuation**  **N=4,104**  **n (%)** | **Switch to NNRTI**  **N=376**  **n (%)** | **Unadjusted**  **OR**  **(95% CI)** | **Adjusted**  **OR**  **(95% CI)** |
| --- | --- | --- | --- | --- | --- |
| Age, median years (IQR) | 4,480 | 44 (40-47) | 35 (30-40) | 1.17 (1.15-1.18) | 1.18 (1.14-1.22) |
| Married or cohabitating | 4,334 |  |  |  |  |
| No |  | 2,228 (54) | 158 (42) | Ref. | Ref. |
| Yes |  | 1,760 (43) | 188 (50) | 0.66 (0.53-0.83) | 0.56 (0.36-0.88) |
| Years in school, median (IQR) | 3,401 | 8 (7-12) | 8 (7-11) | 1.05 (1.00-1.09) | 0.99 (0.92-1.06) |
| Weight, median kg (IQR) | 4,387 | 63 (55-73) | 60 (51-69) | 1.02 (1.01-1.03) | 1.01 (0.99-1.03) |
| WHO stage | 4,183 |  |  |  |  |
| Stage 1 or 2 |  | 2,554 (62) | 216 (57) | Ref. | Ref. |
| Stage 3 or 4 |  | 1,309 (32) | 104 (28) | 1.06 (0.83-1.36) | 0.99 (0.62-1.59) |
| ART regimen at DTG initiation | 4,480 |  |  |  |  |
| Other |  | 433 (11) | 89 (24) | Ref. | Ref. |
| TDF + 3TC + DTG |  | 3,671 (89) | 287 (76) | 2.63 (2.03-3.40) | 3.60 (2.21-5.88) |
| Years on ART, median (IQR) | 4,480 | 7 (4-10) | 5 (1.5-8) | 1.11 (1.08-1.14) | 0.97 (0.90-1.04) |
| Very effective or effective contraception use | 3,983 |  |  |  |  |
| No |  | 1,942 (51) | 65 (45) | Ref. | Ref. |
| Yes |  | 1,895 (49) | 81 (55) | 0.78 (0.56-1.09) | 1.03 (0.66-1.61) |

3TC, lamivudine; ART, antiretroviral treatment; DTG, dolutegravir; IQR, interquartile range; Kg, kilogram; OR, odds ratio; TDF, tenofovir disaproxil; WHO, World Health Organization

**S6 Table.** Sensitivity analysis to determine associations with switch to NNRTI, including (Model A) and excluding (Model B) the variable ‘newly initiated ART’ (n=4,480) and excluding the variable ‘Very effective or effective contraception use’.

Note: This analysis was done because including the “newly initiated ART” variable in the multivariate logistic regression in Table 4 of the main text caused the model to not converge due to numerical considerations (i.e., low number of observations in some cells).

| **Characteristic** | **DTG continuation**  **N=4,104**  **n (%)** | **Switch to NNRTI**  **N=376**  **n (%)** | **Model A**  **Adjusted**  **OR**  **(95% CI)** | **Model B**  **Adjusted**  **OR**  **(95% CI)** |
| --- | --- | --- | --- | --- |
| Age, median years (IQR) | 44 (40-47) | 35 (30-40) | 1.20 (1.17-1.23) | 1.20 (1.17-1.23) |
| Married or cohabitating |  |  |  |  |
| No | 2,228 (54) | 158 (42) | Ref. | Ref. |
| Yes | 1,760 (43) | 188 (50) | 0.58 (0.43-0.79) | 0.58 (0.43-0.78) |
| Years in school, median (IQR) | 8 (7-12) | 8 (7-11) | 0.95 (0.91-1.00) | 0.95 (0.91-1.00) |
| Weight, median kg (IQR) | 63 (55-73) | 60 (51-69) | 1.00 (0.99-1.02) | 1.00 (0.99-1.01) |
| WHO stage |  |  |  |  |
| Stage 1 or 2 | 2,554 (62) | 216 (57) | Ref. | Ref. |
| Stage 3 or 4 | 1,309 (32) | 104 (28) | 0.78 (0.56-1.07) | 0.78 (0.56-1.07) |
| Newly initiated ART |  |  |  |  |
| No | 3,815 (93) | 321 (85) | Ref. | -- |
| Yes | 289 (7) | 55 (15) | 3.15 (0.32-31.01) | -- |
| ART regimen at DTG initiation |  |  |  |  |
| Other | 433 (11) | 89 (24) | Ref. | Ref. |
| TDF + 3TC + DTG | 3,671 (89) | 287 (76) | 3.16 (2.22-4.50) | 3.17 (2.22-4.51) |
| Years on ART, median (IQR) | 7 (4-10) | 5 (1.5-8) | 1.01 (0.96-1.07) | 1.01 (0.96-1.06) |

3TC, lamivudine; ART, antiretroviral treatment; DTG, dolutegravir; IQR, interquartile range; Kg, kilogram; OR, odds ratio; TDF, tenofovir disaproxil; WHO, World Health Organization

**S1 Figure**. Eligibility flow diagram for *Chaguo Langu* study, 2017-2020.

**
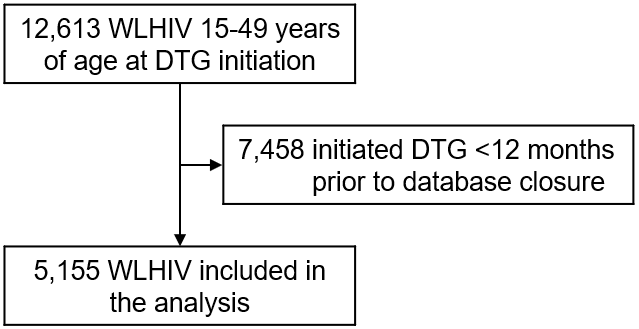
**

**S2 Figure.** Viral suppression among women who continued DTG during the 12 months after DTG initiation.

Key: ART, antiretroviral treatment; DTG, dolutegravir; EFV, efavirenz; NVP, nevirapine

5,155 women included in the analysis

194 excluded

51 with unknown ART regimen prior to DTG initiation

143 transitioned to DTG from regimens not containing EFV or NVP

4,961 transitioned to DTG from regimens containing EFV or NVP (n=4,549), or initiated DTG as part of their first ART regimen (n=412)

253 did not continue DTG for ≥10 weeks

4,708 continued DTG for ≥10 weeks

66 had no viral load result while receiving DTG

4,642 had ≥1 viral load result while receiving DTG

99 had no viral load result ≥10 weeks after DTG initiation

4,543 had ≥1 viral load ≥10 weeks after DTG initiations

**S3 Figure.** Viral suppression among women who switched from DTG to efavirenz or nevirapine during the 12 months following DTG initiation.

Key: ART, antiretroviral treatment; DTG, dolutegravir; EFV, efavirenz; NVP, nevirapine

5,155 women included in the analysis

194 excluded

51 with unknown ART regimen prior to DTG initiation

143 transitioned to DTG from regimens not containing EFV or NVP

4,961 transitioned to DTG from regimens containing EFV or NVP (n=4,549), or initiated DTG as part of their first ART regimen (n=412)

4,463 did not switch to EFV or NVP after initiating DTG

498 switched to EFV or NVP after initiating DTG

36 did not continue EFV or NVP for ≥10 weeks

462 continued EFV or NVP for ≥10 weeks

12 had no viral load result while receiving EFV or NVP

450 had ≥1 viral load result while receiving EFV or NVP

74 had no viral load result ≥10 weeks after receiving EFV or NVP

376 had ≥1 viral load ≥10 weeks after switching to EFV or NVP
